# Sulfonated Poly(Arylene Ether Sulfone) and Perfluorosulfonic Acid Composite Membranes Containing Perfluoropolyether Grafted Graphene Oxide for Polymer Electrolyte Membrane Fuel Cell Applications

**DOI:** 10.3390/polym10060569

**Published:** 2018-05-23

**Authors:** Min-Young Lim, Kihyun Kim

**Affiliations:** 1 Department of Chemical and Biological Engineering and Institute of Chemical Processes, Seoul National University, 599 Gwanak–ro, Gwanak–gu, Seoul 151–744, Korea; mywooya@hanmail.net; 2 Department of Chemistry and Chemical Biology, Rensselaer Polytechnic Institute, 110 8th Street, Troy, NY 12180, USA

**Keywords:** sulfonated poly(arylene ether sulfone), perfluorosulfonic acid, perfluoropolyether grafted graphene oxide, composite membrane, polymer electrolyte membrane fuel cell

## Abstract

Sulfonated poly(arylene ether sulfone) (SPAES) and perfluorosulfonic acid (PFSA) composite membranes were prepared using perfluoropolyether grafted graphene oxide (PFPE-GO) as a reinforcing filler for polymer electrolyte membrane fuel cell (PEMFC) applications. PFPE-GO was obtained by grafting poly(hexafluoropropylene oxide) having a carboxylic acid end group onto the surface of GO via ring opening reaction between the carboxylic acid group in poly(hexafluoropropylene oxide) and the epoxide groups in GO, using 4-dimethylaminopyridine as a base catalyst. Both SPAES and PFSA composite membranes containing PFPE-GO showed much improved mechanical strength and dimensional stability, compared to each linear SPAES and PFSA membrane, respectively. The enhanced mechanical strength and dimensional stability of composite membranes can be ascribed to the homogeneous dispersion of rigid conjugated carbon units in GO through the increased interfacial interactions between PFPE-GO and SPAES/PFSA matrices.

## 1. Introduction

Due to their high energy conversion efficiency and lower environment cost, polymer electrolyte membrane fuel cells (PEMFCs) have received much attention for portable devices, automotives, and residential applications [1]. Among the various components of the PEMFC, the polymer electrolyte membrane (PEM) is regarded as a key component, as it can separate the reactant gases, and provide a pathway for immediate proton transportation [2]. It is generally known that interconnected hydrophilic channels formed by phase separation between hydrophilic and hydrophobic domains in PEMs provide pathways for proton conduction [3]. The most utilized perfluorosulfonic acid (PFSA) membranes, such as Nafion, are composed of a very hydrophobic perfluoro backbone and polar side chains having sulfonic acid groups; the interconnected hydrophilic channels are, therefore, well developed, and can maintain high proton conductivity [4]. However, these PEMs have inherent drawbacks, which include high fuel permeability, low glass transition temperature, and poor thermomechanical properties above 80 °C [5,6]. These drawbacks have prompted the development of alternative PEMs using hydrocarbon-based polymers, including sulfonated poly(arylene ether sulfone)s (SPAES)s, because of their advantages, such as low gas permeability, high thermal stability, and structural diversity [7,8]. However, the high proton conductivity of these PEMs can only be achieved when hydrocarbon-based polymers have a high degree of sulfonation (DS). High DS can develop the interconnected hydrophilic channels, resulting in high proton conductivity [9]. However, the hydrocarbon-based PEMs with high DS do not have high enough dimensional stability for PEMFC operation [10]. One of the effective approaches to improve the dimensional stability of PEMs without much deterioration of the proton conductivity is to incorporate reinforcing inorganic/organic fillers into the polymer matrix [11,12,13].

Recently, carbon nanomaterials, such as carbon nanotube, graphene, and graphene oxide (GO), in the polymer matrix, and their effect on the ion conductivity, mechanical strength, and dimensional stability of the polymer composites, have drawn much attention for scientific research and practical industrial applications [14,15,16]. For example, Jiang et al. presented the construction of tunable ion-conducting nanochannels via direct assembly of GO/poly(phosphonic acid) core–shell nanosheets prepared by surface-initiated precipitation polymerization [17]. The resulting solid electrolyte showed better proton conductivity than commercial Nafion 117 membrane [17]. The remarkable improvement in the mechanical strength and dimensional stability of the polymer composites containing carbon nanomaterials with very small contents is also possible by good interfacial interaction between the fillers and the polymer matrix and well-dispersed state of the fillers. Guiver et al. reported remarkably increased mechanical properties of polymer composites by tuning interfacial interactions between 2D materials (GO and montmorillonite) and polymer matrix [18]. Wang et al. reported an improvement in the mechanical properties and low methanol permeability of hydrocarbon-based PEM by the incorporation of flexible alkylsulfonated grafted GO [19]. Therefore, designing and fine tuning of carbon nanomaterials should be considered to achieve high performances of the polymer composite membranes [20,21,22,23,24].

In this study, we attempted to improve the mechanical and dimensional stability of both hydrocarbon and PFSA-based PEMs without much deterioration of the proton conductivity by using perfluoropolyether grafted GO (PFPE-GO) as a reinforcing filler for the SPAES and Nafion membranes. This paper discusses the detailed preparation methods for the PFPE-GO and composite membranes, including their properties, such as mechanical properties, water absorption behavior, dimensional stability, and proton conductivity.

## 2. Experimental

### 2.1. Materials

Poly(hexafluoropropylene oxide) having a carboxylic acid group located on one chain terminus (DuPont^™^ Krytox^®^, 157 FSL, F–(CF(CF_3_)CF_2_O)_n_–CF(CF_3_) –COOH, *n* = 17, 2500 g/mol) was obtained from DuPont (Wilmington, NC, USA). Graphite powder (Graphite UF 99.5) was received from BASF. 4-Dimethylaminopyridine (DMAP) from Sigma-Aldrich Co., Ltd. (St. Louis, MO, USA) was used as received. Dimethylformamide (DMF), trichlorotrifluoroethane, and ethanol were obtained from Daejung Chemicals & Metals Co., Ltd (Gyeonggi-do, Korea). Nafion (DE 2021, DuPont), a representative ionomer of PFSA, was obtained from Nano Getters Co. (Gyeonggi-do, Korea), as a 20 wt % solution in a mixture of aliphatic alcohols and water. 4,4′-Dichlorodiphenylsulfone (DCDPS, 98.0%, Sigma-Aldrich Co., Ltd.), and 4,4′-dihydroxybiphenyl (BP, 97.0%, Sigma-Aldrich Co., Ltd.) were recrystallized from toluene and methanol, respectively. 3,3′-Disulfonate-4,4′-dichlorodiphenylsulfone (SDCDPS) was synthesized from DCDPS as described by Ueda et al. [25]. The yield of SDCDPS after recrystallization using a mixture of isopropylalcohol and deionized water (7:3, *v/v*) was 83%. All other reagents and solvents were from standard vendors and used as received.

### 2.2. Preparation of GO

Graphene oxide (GO) was synthesized from graphite according to the modified Hummer’s method [20,26], which was described in detail in a previous study [21]. Briefly, a mixture of 1.0 g of graphite powder, 0.5 g of P_2_O_5_, and 6 mL of concentrated sulfuric acid (98.0%) in a reactor was stirred at 80 °C for 6 h, and then 200 mL of deionized water was added into the mixture. The mixture was filtered and washed with deionized water several times. The obtained solid (preoxidized graphite) was dried in a 60 °C vacuum oven for 24 h. The dried solid and 0.5 g of NaNO_3_ were added into a reactor in an ice bath, and 23 mL of concentrated sulfuric acid was added, dropwise, into the reactor without stirring. KMnO_4_ (6.0 g) was added into the mixture, and stirring was maintained at 0–5 °C. The mixture was heated to 35 °C and stirred for 18 h, and then deionized water (200 mL) and H_2_O_2_ (30%, 10 mL) were added. The mixture was centrifuged at 10,000 rpm for 30 min, and the supernatant was decanted. Then, the mixture was filtered through 0.2 μm anodic aluminum oxide membrane filter, and the solid was washed with 250 mL of 10.0% HCl (aq) followed by excessive deionized water until the pH value was 7.0. The resulting product, GO, was dried in an 80 °C vacuum oven for 24 h.

### 2.3. Preparation of Perfluoropolyether-Functionalized GO (PFPE-GO)

GO (0.05 g) was added to 50 mL of DMF and sonicated for 30 min. Krytox^®^ 157 FSL (2.0 g, 0.8 mmol) and DMAP (0.1 g, 0.8 mmol) were added to the GO solution and the reaction mixture was stirred under nitrogen (N_2_) atmosphere at 120 °C for 18 h. The product was obtained by filtering through an anodic aluminum oxide (AAO) membrane filter of 0.2 μm pore size, followed by washing with DMF several times. Then, the product was further purified by dissolving in trichlorotrifluoroethane, filtering, and washing using ethanol to remove any remaining reagents, including Krytox^®^ 157 FSL. The resulting solid (PFPE-GO) was dried overnight in a vacuum oven at 35 °C.

### 2.4. Preparation of Sulfonated Poly(Arylene Ether Sulfone) (SPAES)

SPAES was synthesized by the condensation polymerization of the dihydroxy monomer (BP) with the mixture of DCDPS and SDCDPS as described in our previous report [5]. A 250 mL three-neck round bottom flask equipped with a nitrogen inlet and outlet, a Dean-Stark trap, a condenser, and an overhead mechanical stirrer, was charged with 5.00 g (26.9 mmol) of BP, 3.86 g (13.4 mmol) of DCDPS, 6.60 g (13.4 mmol) of SDCDPS, and 4.27 g (30.9 mmol) of K_2_CO_3_ in 45.2 mL of NMP. Then, 22.6 mL of toluene (NMP/toluene = 2:1, *v/v*) was added as an azeotroping agent. The solution mixture was heated at 145 °C for 4 h to ensure the complete dehydration, and then the temperature was raised to 190 °C for the complete removal of toluene. The reaction was continued for 48 h until the solution became very viscous. The viscous solution was cooled down to room temperature and 10.0 mL of NMP was added to dilute the solution. The solution was filtered to remove the salts and poured into isopropylalcohol (1000 mL) to precipitate the polymer, and then the precipitate was washed several times with isopropylalcohol. The off-white product polymer was obtained in 93% of yield after being dried in a vacuum oven at 60 °C for 24 h. SPAES: ^1^H NMR (DMSO-*d*_6_, 500 MHz): δ 8.31 (br, 2H, ArH), 7.96 (br, 4H, ArH), 7.87 (br, 2H, ArH), 7.73 (br, 8H, ArH), 7.21 (br, 8H, ArH), 7.12 (br, 4H, ArH), 7.03 (br, 2H, ArH).

### 2.5. Preparation of Composite Membranes

The SPAES and Nafion (a representative ionomer of PFSA) composite membranes were fabricated by a typical solution casting method [21]. The following procedure was used for the preparation of SPAES/PFPE-GO-0.1, where 0.1 indicates the weight percent of PFPE-GO to SPAES. The mixture containing 0.5 mg of PFPE-GO in 3.3 g of DMF was sonicated for 30 min and stirred at 60 °C for 1 h to make a homogeneous dispersion of PFPE-GO in DMF. Then, 0.5 g of SPAES powder was mixed with the DMF solution, and the mixture was cast onto a glass plate. The thickness of the cast solution could be controlled using a doctor blade film applicator. The cast solution was then heated in an 80 °C vacuum oven for 12 h. The membrane could be detached by immersing the glass plate in deionized water. The obtained membrane was dried in an 80 °C vacuum oven for 24 h and named as SPAES/PFPE-GO-0.1. Other SPAES/PFPE-GO membranes containing 0.5, 1.0, and 2.0 wt % of PFPE-GO to SPAES were also fabricated, and they were named as SPAES/PFPE-GO-0.5, SPAES/PFPE-GO-1.0, and SPAES/PFPE-GO-2.0, respectively. Nafion composite membranes containing PFPE-GO were also fabricated using the same procedure used for the preparation of the SPAES/PFPE-GO membranes, except for using Nafion instead of SPAES. The Nafion powder was obtained from commercial Nafion solution (20 wt % in a mixture of aliphatic alcohols and water) by evaporating the solvents using rotary evaporator. When 0.1, 0.5, 1.0, and 2.0 wt % of PFPE-GO were incorporated, they were named as Nafion/PFPE-GO-0.1, Nafion/PFPE-GO-0.5, Nafion/PFPE-GO-1.0, and Nafion/PFPE-GO-2.0, respectively. The membranes in salt form were transformed to their respective acid form by soaking in 1 M H_2_SO_4_ aqueous solution at 30 °C for 24 h, and washing with deionized water several times. For comparison, linear SPAES and Nafion membranes were also prepared by the same procedure. The thicknesses of all the membranes were in the range of 30–40 µm.

### 2.6. Characterization

Fourier-transform infrared (FT-IR) spectra of the films were recorded on Cary 660 FT-IR spectrometry (Agilent Technology, Santa Clara, CA, USA) at ambient temperature. Data were collected over 32 scans at 4 cm^−1^ resolution. X-ray photoelectron spectroscopy (XPS) was recorded on a KRATOS AXIS-His (Manchester, UK) using MgKα (1254.0 eV) as the radiation source. Spectra were collected over a range of 0–1200 eV, followed by high resolution scan of the C 1s and F 1s regions. Raman spectra were collected on a T64000 Triple Raman spectrometer (HORIBA, Kyoto, Japan) equipped with a 514.5 nm Ar laser source. Thermal gravimetric analysis (TGA) was performed in a Q-5000 IR (TA Instruments, New Castle, DE, USA). The sample was heated from 25 to 700 °C with a heating rate of 10 °C min^−1^ under a nitrogen atmosphere. The ^1^H NMR spectrum was collected by Varian INOVA-500 NMR spectrometer (Palo Alto, CA, USA) with a proton frequency of 500 MHz. Deuterated dimethylsulfoxide and tetramethylsilane were used as the solvent and the internal standard, respectively. Molecular weights (*M_n_* and *M_w_*) were measured by gel permeation chromatography (GPC, Agilent Technology, Santa Clara, CA, USA), consisting of a Waters 510 HPLC pump at 35 °C, three columns (PLgel 5 μm guard, MIXED-C, MIXED-D), and a Viscoter T60A dual detector. HPLC grade DMF with LiBr was used as the eluent, and the flow rate was 1.0 mL min^−1^. Calibration was conducted by polystyrene standards. Mechanical properties of the membranes were measured using a universal testing machine (Lloyd-LS1, West Sussex, UK). The ASTM standard D638 (Type V specimens) was used for the preparation of dumbbell shape specimens. The measurement was conducted at 25 °C and 40% relative humidity (RH) conditions with a gauge length and cross head speed of 15 mm and 5 mm min^−1^, respectively. For each measurement, at least seven specimens were tested and their average value was calculated. Stress–strain measurements of membranes at different humidity conditions were performed by dynamic mechanical thermal analyzer (TA Q800-RH, TA Instruments, New Castle, DE, USA). Temperature and humidity were controlled in an environmental chamber. The cell temperature and humidity were equilibrated at 50 °C and 50% RH for 40–60 min. Tensile test was conducted using rectangular test strips with membranes. A load ramp 0.5 MPa/min was employed, and each sample was tested twice. The further measurement at 50 °C and 90% RH conditions was conducted by the same methods. The water uptake and dimensional change of the membranes were calculated by measuring their change in weight and volume between the dry and swollen membranes. The dry membranes were cut into 1 cm × 5 cm rectangles, and their weights and volumes were obtained. The membranes were then immersed in deionized water at temperature of 30 °C for 4 h. After the membranes were taken out and wiped with tissue paper, their weights were measured. The change in volume of the membranes was calculated after the membranes immersed in deionized water at 30 °C for 4 h. The water uptake and volume based dimensional change was calculated by the following equations:

Water uptake

[%] = [(*W*_wet_ − *W*_dry_)/*W*_dry_] × 100, (1)

Volume based dimensional change

[%] = [(1 + ΔL)(1 + ΔW)(1+ ΔT) − 1]× 100,(2)

where, *W*_wet_ and *W*_dry_ are the weights of the wet and dry membranes, and ΔL, ΔW, and ΔT are the change of the length, width, and thickness of the membranes, respectively. The oxidative stability of the SPAES composite membranes was estimated by Fenton’s test by calculating the weight change of the samples after being exposed to a Fenton’s reagent (3 wt % H_2_O_2_ aqueous solution containing 4 ppm Fe^2+^). Pre-weighed dry membranes were soaked in a 50 mL of Fenton solution at 80 °C. After 1 h, the membranes were taken out, washed thoroughly with distilled water, and dried in a 60 °C vacuum oven for 24 h before the weight was measured. Hydrolytic stability of the SPAES composite membranes was investigated using a high-pressure chamber filled with deionized water at 100 °C for 24 h. The stability was also evaluated by changes in weight of the membranes. Proton conductivities of the membranes were measured at 80 °C under different relative humidity (RH) conditions using a conductivity measurement system (BekkTech BT-552MX, Loveland, CO, USA) with a H_2_ flow rate of 500 cm^3^ min^−1^. The samples were pre-equilibrated at 80 °C and 70% RH for 2 h, and then the conductivity measurements were performed. The equilibrium RH was obtained after about 15 min of stabilization time.

## 3. Results and Discussion

### 3.1. Preparation and Characterization of PFPE-GO

**Figure 1:**
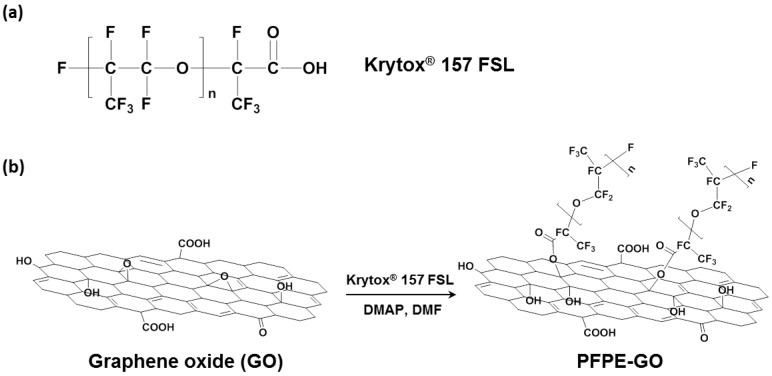
Chemical structure of (**a**) Krytox^®^ 157 FSL and schematic diagram of (**b**) the preparation of perfluoropolyether grafted graphene oxide (PFPE-GO).

Perfluoropolyether-functionalized GO, named as PFPE-GO, was prepared from the reaction of GO with a commercially available poly(hexafluoropropylene oxide) having a carboxylic acid group, Krytox^®^ 157 FSL (Figure 1a), in the presence of a base catalyst, DMAP, as shown in Figure 1b; the carboxylic acid group in Krytox^®^ 157 FSL was reacted with the epoxide groups in GO via a base-catalyzed ring opening reaction [27,28].

**Figure 2:**
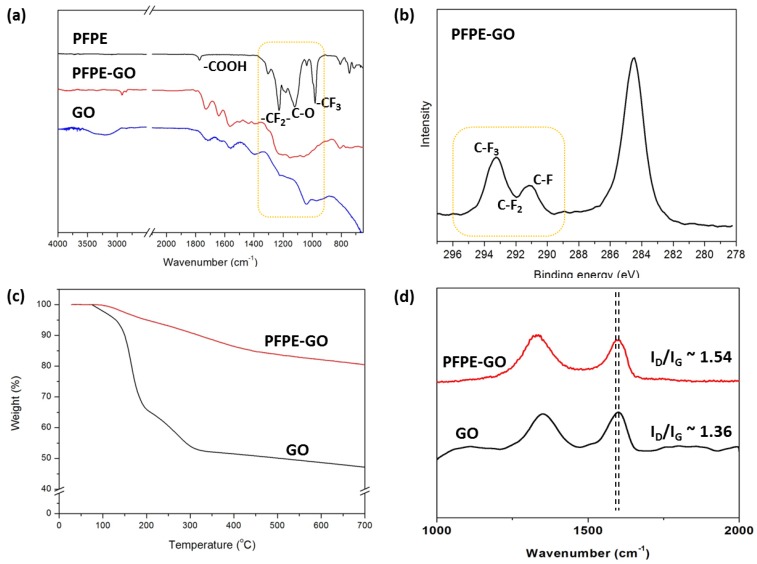
(**a**) FT-IR spectra of PFPE, GO, and PFPE-GO, (**b**) XPS spectrum in the C 1s region for PFPE-GO, (**c**) TGA curves of GO and PFPE-GO, and (**d**) Raman spectra of GO and PFPE-GO.

Figure 2a shows the FT-IR spectra of GO, Krytox^®^ 157 FSL, and PFPE-GO. In the spectrum of GO, a broad O–H stretching peak from the hydroxyl groups and the water adsorbed in GO at 3450 cm^−1^, a C=O peak from the ketone and carboxyl acid groups at 1740 cm^−1^, aromatic C=C and O–H bending peaks from phenolic groups at 1620 cm^−1^, a C–O peak from the epoxy groups at 1240 cm^−1^, and a C–O peak in the alkoxy groups at 1050 cm^−1^ are observed [15,21]. On the contrary, the IR spectrum of PFPE-GO shows characteristic absorption peaks at 1153, 1200, and 1230 cm^−1^ corresponding to –CF_2_– stretching, and a weak absorption peak at 980 cm^−1^ corresponding to –CF_3_ stretching from the fluoropropylene oxide groups in the PFPE backbone, indicating the incorporation of the PFPE moieties in GO [29]. The C1s XPS spectrum of PFPE-GO also indicates the incorporation of the PFPE moieties in GO to form PFPE-GO (Figure 2b). The C1s XPS spectrum of GO shows four peaks from C–C, C–O, C=O, and O–C=O at 284.5, 286.5, 287.5, and 288.5 eV, respectively [30,31,32], while that of PFPE-GO shows new peaks at higher boding energies, such as 293.3 eV, 291.8 eV, and 291.3 eV attributed to carbon in –CF_3_, –CF_2_–, and –CF groups of PFPE, indicating the successful incorporation of the PFPE group into GO to form PFPE-GO [25,33]. The existence of grafted PFPE moieties on GO sheets could be also confirmed by the F1s XPS spectrum of PFPE-GO (Figure S1). The F1s peak at 689.0 eV clearly indicates the existence of fluorine element in PFPE-GO.

The amount of the grafted PFPE moiety on GO was estimated from the TGA curves for GO and PFPE-GO as shown in Figure 2c. It is well known that the weight change below 300 °C is due to the thermal reduction of the oxygen functional groups in GO, and that between 300 and 500 °C is due to the thermal decomposition of the other organic moieties in GO [20,34,35]. Therefore, 8 wt % of the weight loss between 300–500 °C for PFPE-GO indicates the approximate amount of the PFPE moiety grafted on GO. The smaller weight loss of PFPE-GO below 300 °C than that of GO indicates that PFPE-GO contains less oxygen functional groups than GO. Since PFPE-GO was prepared at high temperature (120 °C), it is possible that the oxygen functional groups in GO could be reduced. The reduction was further confirmed by the red shift of 10 cm^−1^ in Raman spectra from 1603 cm^−1^ of the G band for GO to 1593 cm^−1^ for PFPE-GO (Figure 2d), as reported previously [36,37]. Moreover, the C1s XPS spectrum of PFPE-GO also indicates that PFPE-GO is reduced; the relative peak intensity of the C–O groups in the C1s XPS spectrum of PFPE-GO is considerably smaller than that of GO because the epoxide groups in GO were removed by the reaction with the carboxylic acid group in Krytox^®^ 157 FSL.

### 3.2. Synthesis of Sulfonated Poly(Arylene Ether Sulfone) (SPAES)

**Figure 3:**
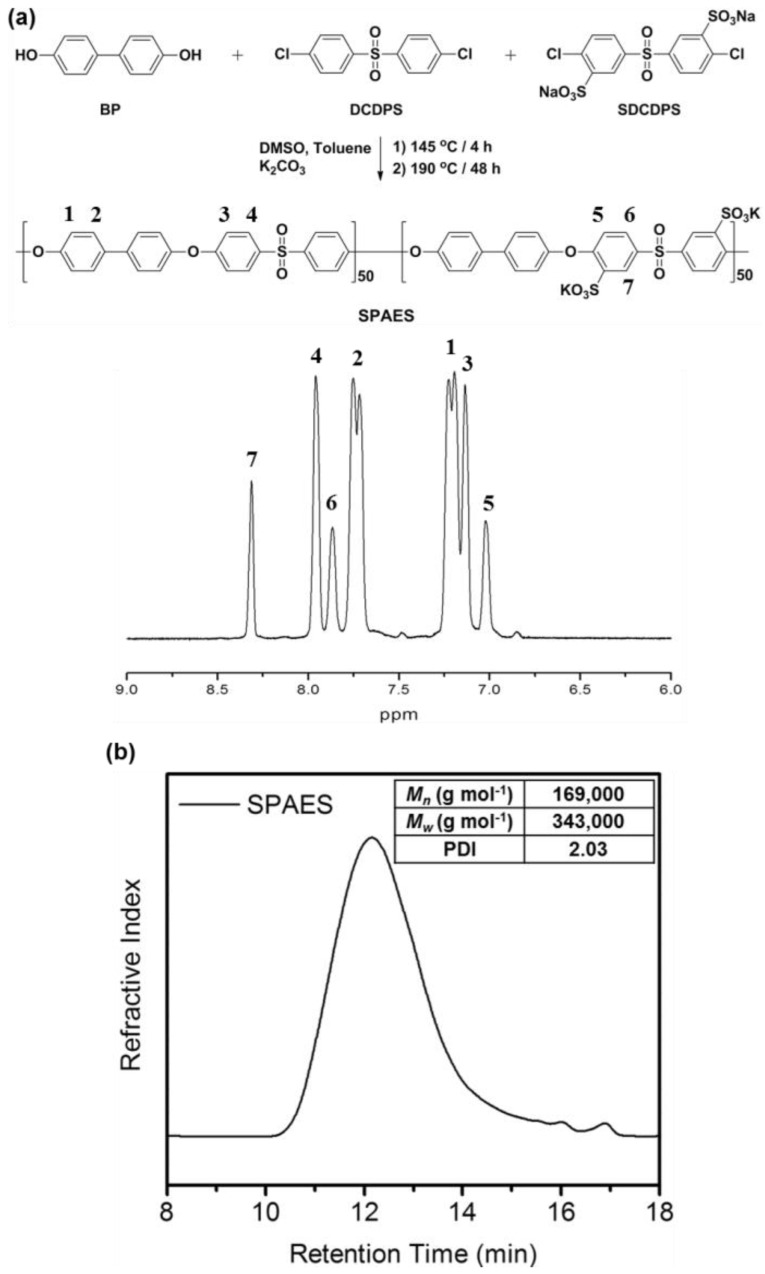
(**a**) Synthesis scheme and ^1^H NMR spectrum, and (**b**) GPC profile of sulfonated poly(arylene ether sulfone) (SPAES).

Figure 3a shows that SPAES in potassium salt form (K^+^) was synthesized via nucleophilic step-growth polymerization using the monomer mixture of BP, DCDPS, and SDCDPS [38]. The chemical structure and composition of the obtained SPAES were confirmed by ^1^H NMR spectroscopy (Figure 3a). Since the feed monomer ratio of sulfonated (SDCDPS) and non-sulfonated (DCDPS) dichloro monomers is 1:1, degree of sulfonation (DS, mol %) of 50 was expected, while the DS of SPAES measured by the peak integration from the ^1^H NMR spectrum was about 47. It is possible a slightly different reactivity between SDCDPS and DCDPS induced by different chemical structure caused this result [39]. SPAES with DS of about 50 was intentionally prepared to fabricate linear and composite membranes in this study, because SPAES with a DS larger than 50 has been known to not have sufficient dimensional stability to provide membrane stability for intermediate temperature PEMFCs, based on our previous studies [4,5]. The number average molecular weight (*M_n_*) and weight-average molecular weight (*M_w_*) calculated from the result of GPC measurement using polystyrene standards were 169,000 and 343,000, respectively (Figure 3b).

### 3.3. Mechanical Properties

**Figure 4:**
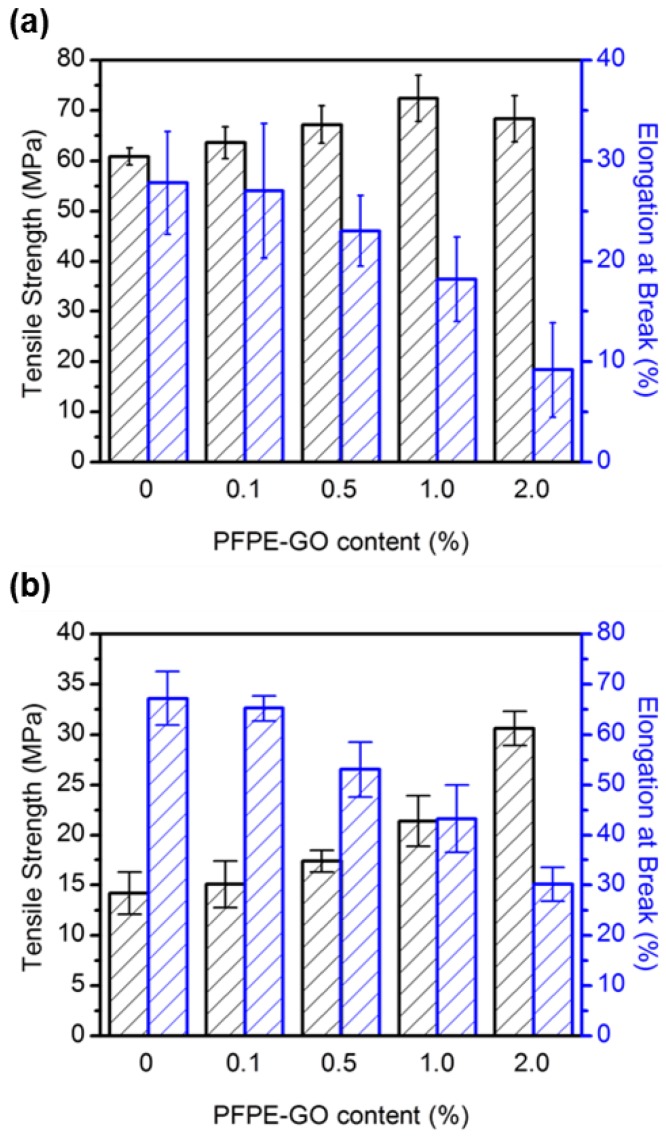
Mechanical properties of (**a**) SPAES/PFPE-GO and (**b**) Nafion/PFPE-GO membranes.

During the fuel cell operation, cracks and pinholes of the PEMs resulted in increased fuel crossover, which reduces cell performance and accelerates degradation. Mechanical degradation of the membrane electrode assemblies can be mitigated through the use of high tensile strength membranes with high elongation at break [40,41]. Figure 4 and Table 1 show the mechanical properties of the membranes, such as tensile strength, and elongation at break, estimated by a universal testing machine (UTM) under room temperature and 45% relative humidity (RH) conditions. It is well known that the incorporation of inorganic fillers in polymer matrix can enhance the mechanical strength (i.e., tensile strength) by the reinforcement effect of the fillers, if the fillers are not phase-separated [42,43]. The addition of PFPE-GO was found to improve the mechanical strength of the SPAES and Nafion membranes. For example, the tensile strength value of SPAES increased from 60.8 to 72.4 MPa by adding 1.0 wt % of PFPE-GO and that of Nafion also increased from 14.2 to 30.6 MPa by adding 2.0 wt % of PFPE-GO. The mechanical reinforcement of the composite membranes can be ascribed to 1) the incorporation of rigid conjugated carbon units by adding PFPE-GO, and 2) the increase in the stiffness of the polymer chain, because the PFPE-GO sheets can restrict the stretching of the polymer chain through the interaction between the oxygen functional groups in GO and the sulfonic acid groups in both SPAES and Nafion [21,44,45]. Although the tensile strength values of the SPAES/PFPE-GO membranes are larger than those of the Nafion/PFPE-GO membranes, the reinforcing efficiency of PFPE-GO in Nafion is better than that in SPAES, due to the better dispersion state of PFPE-GO having perfluoro moieties. Since the phase separation of the PFPE-GO domains in the polymer matrix can deteriorate the mechanical properties [12,15,46], the tensile strength of the SPAES/PFPE-GO decreases when the PFPE-GO content is 2.0 wt %. The dispersion state of PFPE-GO in SPAES matrix was confirmed by the cross-sectional SEM image of the cryo-fractured SPAES/PFPE-GO membranes (Figure S2). Meanwhile, the elongation at break values of all the composite membranes are lower than those of each linear SPAES and Nafion membrane, and the values are decreased with the increase in PFPE-GO contents, as reported by the other composite membranes containing reinforcing fillers, such as carbon nanomaterials [20,47,48]. It is generally known that mechanical properties of hydrocarbon-based PEMs are prone to humidity change, due to their inadequate phase-separated structure between hydrophilic and hydrophobic domains, compared to the PFSA membranes [9]. Therefore, it is desirable to confirm the change of mechanical properties under different humidity conditions. The mechanical properties of SPAES and SPAES/PFPE-GO-1.0, a representative sample of the SPAES-based composite membranes, were measured using DMA at 50 °C and different RH conditions of 50% and 90% RH. As shown in Figure S3, the difference of both stress and elongation at break between SPAES and SPAES/PFPE-GO-1.0 membranes is increased by the increase of RH, due to the reinforcing effect of PFPE-GO and low water absorption behavior of the SPAES/PFPE-GO-1.0 membrane compared to the SPAES membrane, as described in next section.

### 3.4. Water Uptake and Dimensional Change

The water absorption and dimensional stability behaviors of the membranes were estimated by measuring their water uptake and dimensional change after being soaked in deionized water at 30 °C for 24 h, respectively (Table 1). The water uptakes of the SPAES/PFPE-GO and Nafion/PFPE-GO membranes were found to be lower than those of the linear SPAES and Nafion membranes, respectively, and the values decreased with the increase in the PFPE-GO content. For example, the water uptake values of the SPAES/PFPE-GO-0.5 and Nafion/PFPE-GO-0.5 membranes were 64.3% and 30.8%, respectively, while those of the linear SPAES and Nafion membranes were 66.7% and 35.6%, respectively. The increase in the PFPE-GO content from 0.1 to 2.0 wt % further decreased the water uptake values from 66.0% to 55.4% for the SPAES/PFPE-GO membranes and from 34.2 to 23.3% for the Nafion/PFPE-GO membranes, because the increase in the interfacial area between the polymer matrix (SPAES or Nafion) and the PFPE-GO having perfluoro polymer chains further restricts the chain motion and the free volume of polymer for water storage [49]. For example, in the case of SPAES/PFPE-GO, the entanglement interaction between the flexible perfluoroalkyl chains grafted in PFPE-GO and the SPAES backbone as well as the interaction between oxygen functional groups in GO and the sulfonic acid groups in SPAES affect the water absorption behavior [19].

The dimensional change behavior of the membranes is similar to the water absorption behavior, because it is strongly associated with water content of the membrane [8,50]. Table 1 shows the dimensional change values of the membranes after being immersed in deionized water at 30 °C for 24 h. The SPAES/PFPE-GO and Nafion/PFPE-GO membranes exhibit much improved dimensional stability and the values were found to be affected by the content of PFPE-GO; the volume change values of the SPAES/PFPE-GO-0.1 and −1.0 membranes were 56.2% and 45.7%, respectively, and those of the Nafion/PFPE-GO-0.1 and −1.0 membranes were 45.0% and 34.8%, respectively, while those of the linear SPAES and Nafion were 63.0% and 47.1%, respectively. Although the decrement of the dimensional change and water uptake values of each SPAES/PFPE-GO and Nafion/PFPE-GO membranes were different because Nafion and SPAES have different polymer structures and ion exchange capacities, these results clearly indicate that the incorporation of PFPE-GO having hydrophobic perfluoro chain can effectively suppress the excessive water absorption, and improve the dimensional stability of both hydrocarbon and PFSA-based PEMs. Oxidative and hydrolytic stabilities of PEMs are useful parameters that can be estimate the long-term durability of PEMFCs [19]. The oxidative and hydrolytic stabilities of the SPAES/PFPE-GO membranes are also shown in Table S1.

### 3.5. Proton Conductivity

**Figure 5:**
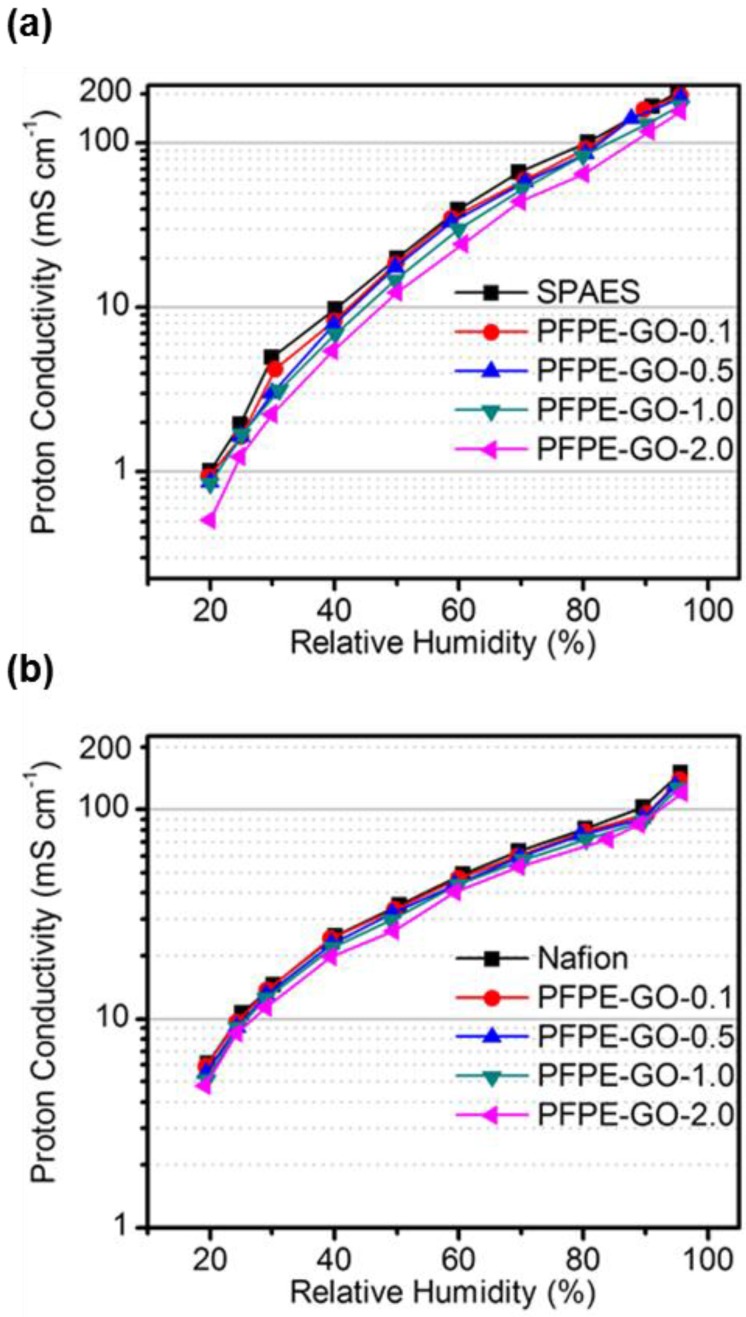
Proton conductivities of (**a**) SPAES/PFPE-GO and (**b**) Nafion/PFPE-GO membranes at 80 °C as a function of relative humidity.

Figure 5a,b show the proton conductivities of the membranes measured at 80 °C under different RH conditions from 20% to 90%. As the RH increases, the proton conductivity of all the membranes increases, because the amount of absorbed water by the hydrophilic channels increases [1]. The proton conductivity values of all the SPAES and Nafion composite membranes were found to be comparable to, or slightly lower than those of each linear SPAES and Nafion membranes. This is possibly because the PFPE-GO sheets work as a barrier that can disconnect the hydrophilic channels and consequently restrict the proton conduction. Although some studies reported that hydrophilic oxygen functional groups in graphene oxide participate in proton conduction by forming a hydrogen-bonded network using absorbed water, which increase the proton conductivity [21,51], our composite membranes exhibit slightly lower proton conductivities because lots of oxygen functional groups in GO were reduced during the preparation of PFPE-GO, as described in previous part. Therefore, the barrier effect of the PFPE-GO sheets more dominantly affected the proton transfer through the hydrophilic channels, even though a small amount of oxygen functional groups remained in PFPE-GO. Others also reported that reduced graphene oxide and graphene sheets can disconnect the hydrophilic channels of proton exchange membranes [52,53,54]. However, the proton conductivity values of the Nafion/PFPE-GO membranes were found to be almost comparable to those of the Nafion membrane, even though in high RH conditions (RH 70–95%), as shown in Figure 5b. This result could be ascribed to the combined effect of good dispersion of PFPE-GO in the Nafion matrix having similar perfluoro structure that could possibly minimize the barrier effect of the filler by aggregation, and also decrease the dissolution of ionic concentration of the membranes by preventing excessive swelling in high RH conditions. Similar proton conducting behavior of the SPAES/PFPE-GO membranes could be observed when the PFPE-GO content was smaller than 1.0 wt % (Figure 5a). However, when the PFPE-GO content was larger than 1.0 wt %, the proton conductivity difference between the SPAES and SPAES/PFPE-GO membranes increase because the PFPE-GO was not effectively dispersed by the phase separation, which normally deteriorates the membrane properties [12].

## 4. Conclusions

Sulfonated poly(arylene ether sulfone) (SPAES) and Nafion (a representative ionomer of perfluorosulfonic acid (PFSA)) composite membranes containing perfluoropolyether grafted graphene oxide (PFPE-GO) were prepared for polymer electrolyte membrane fuel cell applications. Although the proton conductivity of the SPAES and Nafion composite membranes are comparable to, or slightly lower than each linear Nafion and SPAES membrane, due to the combined effect of hydrophobic nature of PFPE and reduced GO, the incorporation of PFPE-GO having perfluoropolyether groups is found to be an effective strategy to improve both mechanical strength and dimensional stability of the membranes. Adding a small amount of PFPE-GO was found to improve the mechanical strength and dimensional stability of the SPAES/PFPE-GO and Nafion/PFPE-GO composite membranes. This could be ascribed to the organic (perfluoropolyether) moieties in GO increasing the interfacial interactions between the fillers and the polymer matrices (SPAES/Nafion), then the homogeneously-dispersed rigid conjugated carbon units in GOs are able to increase the mechanical strength and dimensional stability of the membranes. We believe that PFPE-GO was an effective filler material to increase the interactions with both hydrocarbon and PFSA-based PEMs, to simultaneously improve the mechanical strength and dimensional stability without much deterioration of their proton conductivity.

## Figures and Tables

**Table 1:** Mechanical properties, water uptake and swelling ratio of membranes.

Membrane	Tensile Strength (MPa)	Elongation at Break (%)	Water Uptake (%)	Swelling Ratio (%)	
				ΔArea	ΔVolume
SPAES	60.8 ± 1.7	27.8 ± 5.1	66.7	32.6	63.0
SPAES/PFPE-GO-0.1	63.6 ± 3.2	27.0 ± 6.7	66.0	27.7	56.2
SPAES/PFPE-GO-0.5	67.2 ± 3.7	23.0 ± 3.5	64.3	27.3	53.0
SPAES/PFPE-GO-1.0	72.4 ± 4.6	18.2 ± 4.2	55.4	21.7	45.7
SPAES/PFPE-GO-2.0	68.4 ± 4.6	9.2 ± 4.7	56.7	24.3	48.6
Nafion	14.2 ± 2.1	67.2 ± 5.3	35.6	25.4	47.1
Nafion/PFPE-GO-0.1	15.1 ± 2.3	65.2 ± 2.5	34.2	23.5	45.0
Nafion/PFPE-GO-0.5	17.4 ± 1.1	53.0 ± 5.5	30.8	21.6	40.1
Nafion/PFPE-GO-1.0	21.4 ± 2.5	43.2 ± 6.7	27.3	17.3	34.8
Nafion/PFPE-GO-2.0	30.6 ± 1.7	30.2 ± 3.4	23.3	15.2	30.2

## Supplementary Materials

The following supplementary materials are available online at http://www.mdpi.com/2073-4360/10/6/569/s1.
