# Supplementary material for: Sulfonated Poly(Arylene Ether Sulfone) and Perfluorosulfonic Acid Composite Membranes Containing Perfluoropolyether Grafted Graphene Oxide for Polymer Electrolyte Membrane Fuel Cell Applications
Source: Polymers (Basel). 2018 May 23;10(6):569. doi: 10.3390/polym10060569 (PMC6403734; doi:10.3390/polym10060569)
Supplement: Supplementary file 1 [file polymers-10-00569-s001.pdf]

Supporting Information

Sulfonated Poly(Arylene Ether Sulfone) and Perfluorosulfonic Acid Composite Membranes Containing Perfluoropolyether Grafted Graphene Oxide for Polymer Electrolyte Membrane Fuel Cell Applications

*Min-Young Lim[a], and Kihyun Kim[b]**

[a] Department of Chemical and Biological Engineering and Institute of Chemical Processes, Seoul National University, 599 Gwanak–ro, Gwanak–gu, Seoul 151–744, Republic of Korea

[b] Department of Chemistry and Chemical Biology, Rensselaer Polytechnic Institute, 110 8th Street, Troy, New York 12180, United States

*Corresponding Author: Tel. +1 518 833 2844; e-mail: kimk14@rpi.edu

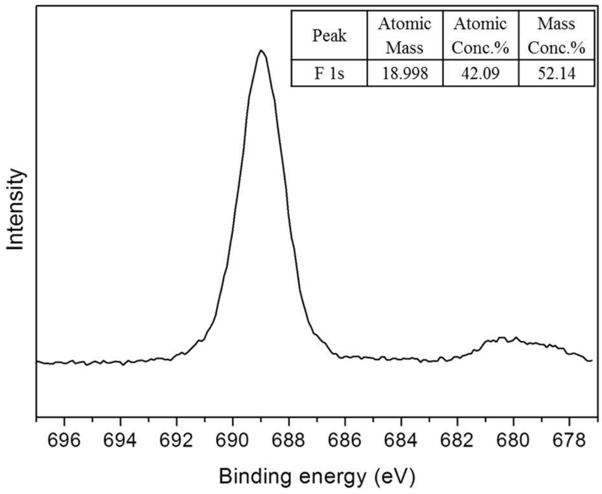

Figure S1. XPS spectrum in the F1 region for PFPE-GO.

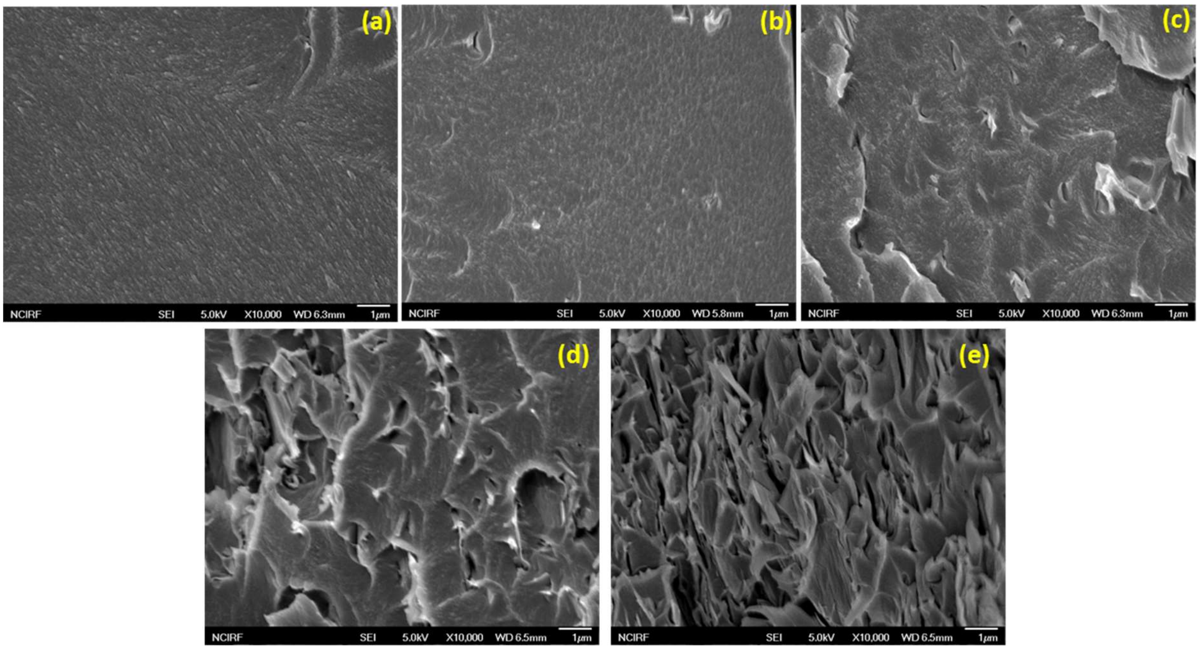

Figure S2. Cross-sectional SEM images of SPAES and composite membranes: (a) SPAES, (b) SPAES/PFPE-GO-0.1, (c) SPAES/PFPE-GO-0.5, (d) SPAES/PFPE-GO-1.0 and (e) SPAES/PFPE-GO-2.0.

The cross-sectional morphology of the SPAES/PFPE-GO membranes was analyzed by JSM-6700F (JEOL, Japan) using a field emission scanning electron microscope (FE-SEM). The samples for the FE-SEM measurements were prepared by cutting the membranes after quenching using liquid nitrogen.

The SPAES membrane shows a relatively uniform and smooth surface without any pinholes, while as the filler content increases, the composite membranes show much rougher surface images with wrinkles and/or agglomerated domains due to the mutual interaction between the SPAES matrix and the GO derivatives [1,2]. The agglomerated domains are observed from

the SPAES/PFPE-GO membranes when the filler contents are larger than 1.0 wt.% due to the poor dispersion of the PFPE-GO nanosheets.

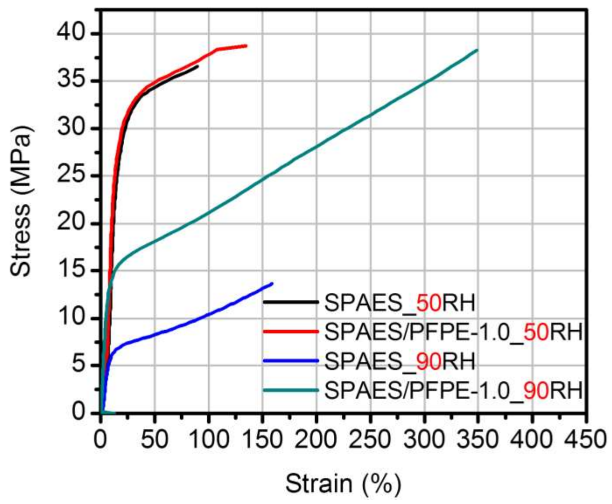

Figure S3. Stress versus strain curves of SPAES and SPAES/PFPE-GO-1.0 membranes at 50 °C under different humidity conditions of 50% and 90% RH.

Table S1. Oxidative and hydrolytic stability of SPAES and composite membranes.

Membrane	Residual weight[a] (%)	Residual weight[b] (%)
SPAES	82	100
SPAES/PFPE-GO-0.1	83	100
SPAES/PFPE-GO-0.5	88	100
SPAES/PFPE-GO-1.0	85	100
SPAES/PFPE-GO-2.0	80	100

[a] Obtained by Fenton's test.

[b] Obtained by hydrolytic stability test.

The oxidative stability of the membranes was investigated by measuring the residual weight of the membranes after 1 h of immersion in Fenton's reagent (3 wt.% H_2O_2 aqueous solution containing 4 ppm Fe^{2+}) at 80 °C. Table S1 shows that the composite membranes are more stable than the linear SPAES membrane when the PFPE-GO contents are smaller than 1.0 wt.%. The oxidative stability of the SPAES/PFPE-GO membranes initially increases with the PFPE-GO content due to the radical scavenging ability of GO [3,4]. However, when the PFPE-GO content is larger than 1.0 wt.%, the oxidative stability of the SPAES/PFPE-GO decreases due to the possible phase separation of the GO domains in the SPAES matrix that can decrease the radical scavenging ability of GO in the polymer matrix and due to the defect structures formed in the polymer that can further deteriorate the oxidative stability.

The hydrolytic stability of the membranes was investigated by changes in weight of the membranes after immersion in deionized water at 100 °C for 24 h. The SPAES composite membrane as well as the linear SPAES membrane exhibit negligible changes in weight

because of relatively low ion exchange capacity (1.97 mequiv. g^{-1}) and well-known hydrolytic stability of SPAES [5].

References

[1] T. Ko, K. Kim, M.Y. Lim, S.Y. Nam, T.H. Kim, S.K. Kim, J.C. Lee, Sulfonated poly(arylene ether sulfone) composite membranes having poly(2,5-benzimidazole)-grafted graphene oxide for fuel cell applications, *J. Mater. Chem. A*, **2015**, 3, 20595-20606.

[2] L. Zhao, Y. Li, H. Zhang, W. Wu, J. Liu, J. Wang, Constructing proton-conductive highways within an ionomer membrane by embedding sulfonated polymer brush modified graphene oxide, *J. Power Sources*, **2015**, 286, 445-457.

[3] K. Kim, J. Bae, M.-Y. Lim, P. Heo, S.-W. Choi, H.-H. Kwon, J.-C. Lee, Enhanced physical stability and chemical durability of sulfonated poly(arylene ether sulfone) composite membranes having antioxidant grafted graphene oxide for polymer electrolyte membrane fuel cell applications, *J. Membr. Sci.*, **2017**, 525, 125-134.

[4] Y. Qiu, Z. Wang, A.C. Owens, I. Kulaots, Y. Chen, A.B. Kane, R.H. Hurt, Antioxidant chemistry of graphene-based materials and its role in oxidation protection technology, *Nanoscale*, **2014**, 6, 11744-11755.

[5] M.A. Hickner, H. Ghassemi, Y.S. Kim, B.R. Einsla, J.E. McGrath, Alternative polymer systems for proton exchange membranes (PEMs), *Chem. Rev.*, **2004**,104, 4587-4612.
